# Long‐term drinking of green tea combined with exercise improves hepatic steatosis and obesity in male mice induced by high‐fat diet

**DOI:** 10.1002/fsn3.3773

**Published:** 2023-12-21

**Authors:** Ruru Wang, Mingxing Gu, Yanzhong Zhang, Qinglin Zhong, Linbo Chen, Daxiang Li, Zhongwen Xie

**Affiliations:** ^1^ State Key Laboratory of Tea Plant Biology and Utilization, School of Tea and Food Sciences and Technology Anhui Agricultural University Hefei China; ^2^ Department of Sports Sciences Anhui Agricultural University Hefei China; ^3^ Tea Research Institute Yunnan Academy of Agricultural Sciences Kunming China

**Keywords:** hepatic steatosis, inflammation, NF‐κB, obesity, treadmill exercise, Yunkang 10 green tea (GT)

## Abstract

Dietary habits and exercise play an important role in the well‐being of human health. Currently, how long of drinking tea combined with exercise could efficiently ameliorate hepatic steatosis and obesity still needs to be investigated. Here, short‐term and long‐term green tea drinking combined with exercise were studied to improve hepatic steatosis and obesity in high‐fat diet‐induced (HF) mice. Our results showed that Yunkang 10 green tea (GT) combined with exercise (Ex) exhibited synergistic prevention effects on ameliorating hepatic steatosis and obesity. Especially, 22‐week intervention with GT or Ex improved all symptoms of obesity, which indicated that long‐term intervention exhibited profound preventive effects than the short term. Moreover, the combined intervention of 22 weeks inhibited the activation of NF‐κB pathway and the expression of proinflammatory cytokines, which suggests that tea combined exercise may improve liver steatosis mainly by inhibiting inflammation. The key molecules for regulating lipid and glucose metabolism SCD1 were obviously downregulated, and GLU2 and PPARγ were significantly upregulated by GT and exercise in the liver of high‐fat diet‐induced mice. This study demonstrated that long‐term intervention with GT and exercise effectively relieved hepatic steatosis and obesity complications by ameliorating hepatic inflammation, reducing lipid synthesis, and accelerating glucose transport.

## Graphical abstract

1. Long‐term intervention with YKGT and exercise effectively relieved hepatic steatosis and obesity complications by ameliorating hepatic inflammation, reducing lipid synthesis, and accelerating glucose transport.
2. Long‐term intervention exhibited profound beneficial effects than short term on ameliorating symptoms of hepatic steatosis and obesity complications.
3. The key molecules, SCD1, GLU 2, and PPARγ, were identified for playing important roles in regulating lipids and glucose homeostasis upon YKGT and exercise intervention.

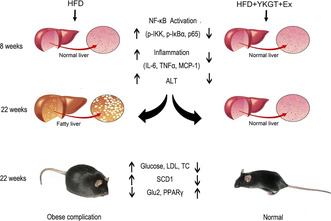

## 1 INTRODUCTION

Dietary habits and exercise play an important role in human health. Currently, how long drinking tea with exercise could efficiently ameliorate hepatic steatosis and obesity‐induced adverse reactions still needs to be investigated. Hepatic steatosis associated with obesity is the most common cause of chronic liver disease and may progress to nonalcoholic steatohepatitis and end‐stage liver disease (Sheka et al., 2020). The risk of developing hepatic steatosis is closely related to diet and lifestyle. The rapid development of the economy has led to an excessive supply of food. At the same time, with the development of technology, people's physical activity is reduced, which is more likely to cause obesity. (Moon et al., 2017; Swinburn et al., 2011). Nowadays, sedentary lifestyles and overeating habits have been the primary causes of the growing rate of obesity across the world (Blueher, 2019; Hossain et al., 2007; Mozaffarian et al., 2011). Although great progress has been made in the treatment of obesity, there are still some potential risks. (Blueher, 2019; Inge et al., 2016). At present, improving unhealthy lifestyle, reducing caloric intake, and moderate exercise are the basic strategies to prevent obesity. Regular exercise can improve the body's oxidative capacity and lipid metabolism and reduce obesity indicators (Roberts et al., 2013; Wood et al., 2021). However, how long the combined intervention of tea drinking and exercise could perform profound beneficial effects and the underlying molecular mechanism need to be further investigated.

Tea is one of the most popular beverages because of its natural and healthy properties. Green tea contains a large amount of single catechin, and (−)‐epigallocatechin gallate (EGCG) is the main component of catechin. Many reports have proved that catechins have good functions in promoting fat oxidation and reducing total cholesterol and triglycerides in blood (Yang et al., 2016). Yun Kang 10 (*Camellia sinensis var. assamica* cv. Yun Kang 10) is the main cultivated tea cultivar in Southwestern China. The chemical profiling and volatile components of Yun Kang 10 have been reported (Wang et al., 2017). In a previous paper, our group reported that Yunkang 10 green tea has a higher amount of monomeric catechin, and a combination of Yunkang 10 green tea supplementation with aerobic exercise synergistically ameliorated the existing metabolic syndrome (Zhang et al., 2020). Previous studies have also shown that drinking green tea can help increase the calories consumed during exercise and effectively prevent obesity (Sae‐Tan et al., 2015; Shimotoyodome et al., 2005). This study aims to investigate whether a combination of Yunkang 10 green tea and treadmill exercise exhibits synergistically preventive effects and to compare how long intervention period (8 weeks vs. 22 weeks) will show effective preventive effects on hepatic steatosis and obesity induced by HFD in C57BL/6J mice.

## 2 MATERIALS AND METHODS

### 2.1 Tea sample

Yunkang 10 green tea was produced by steaming the leaves collected from Yun Kang 10 tea trees (*Camellia sinensis var. assamica* cv. Yun Kang 10) in Menghai County of Yunnan province, China following a standard protocol. BLENDER 800S (Warning, Corp., Torrington, CT, USA) was used to crush Yunkang 10 green tea into powder and then powder was added to HFD at a concentration of 5% (w/w). The contents of catechin and caffeine were detected by high‐performance liquid chromatography (HPLC). The instrument uses a waters 600E high‐performance liquid chromatograph, selects a phenomenex Gemini C18 column for separation, and detects and analyzes at 280 nm. The detailed contents of catechin and caffeine in Yunkang 10 green tea are shown in Table 1.

**TABLE 1:** Contents of catechin and caffeine in Yunkang 10 green tea.

Composition	Content (%)	Composition	Content (%)
Caffeine	4.051 ± 0.306	+C	0.252 ± 0.064
EGCG	12.500 ± 1.048	EC	0.668 ± 0.038
ECG	3.282 ± 0.272	GCG	0.229 ± 0.020
EGC	1.439 ± 0.092	Total Catechins	18.370 ± 1.524

### 2.2 Animal experiments and ethics approval

The specific pathogen‐free male C57BL/6J mice, the age of 5 weeks and the weight of 18–21 g, were purchased from Vital River Laboratory Animal Technology Co., Ltd. (Beijing, China). The animals were housed in cages at the Animal Facility Center of Anhui Agricultural University, which was controlled with constant temperature (22 ± 1°C) and humidity (50 ± 5%) with a 12:12 h light–dark cycle falls at 8:00 a.m. to 8:00 p.m. The mice were provided with standard AIN93 food and water ad libitum. After 3 weeks of acclimation, the mice were fed a low‐fat diet (LF, TP23303, 11% of energy derived from fat), high‐fat diet (HF, TP23300, 60% of energy derived from fat), HF with 5% GT (GT), HF with treadmill exercise (Ex), HF with GT plus Ex (GT + Ex), respectively. Each group had 12 mice. All diets were obtained from Trophic Animal Feed High‐tech Co., Ltd. (Nantong, China). The composition of the diets is listed in Supplementary Table S1. The exercise mice received treadmill running 6 days per week during the experimental period. A detailed treadmill running schedule is provided in Supplementary Table S2. This study adds tea powders to diet for free consumption by mice. Food intake and water consumption were recorded every day. Body weight was monitored with a weight scale weekly.

### 2.3 Serum and tissue sample collection

After the experiment, the mice fasted for 24 h and were anesthetized with 4% chloral hydrate (10 mL/kg, i.p.). Peripheral blood was collected from the eye vein to obtain serum and then stored at −80°C. Quickly mouse liver and abdominal adipose tissue were taken out, weighed and recorded the weight, and then a quarter of the tissue was saved in RNA stable solution (Thermo Fisher Scientific, Baltics, USA) for gene expression analysis, and a quarter of the tissue in formaldehyde solution (Zhangyun, Jiangsu, China) for histological experiment. The rest tissues were immediately frozen in liquid nitrogen and stored at −80°C for protein expression research.

### 2.4 Serum biochemical parameter analysis

The content of LDL and TC in serum was measured with a microtest kit (Johnson medical equipment, Shanghai, China). The enzyme kit (Jiancheng Biotechnology, Nanjing, China) was used to analyze the enzyme activity of ALT in serum. The fasting blood glucose of mice was measured with Nova StatStrip XpresstM Glucose CR instrument (Nova Biomedical, Waltham, UK) and Nova StatStrip XpresstM‐Glu test Strip (Nova Biomedical, Waltham, UK).

### 2.5 Quantitative real‐time PCR assay

SYBR Green Master Mix (CFX96 Touch, Bio‐RAD, USA) was used for real‐time PCR according to the previous protocol (Xie et al., 2011). Primer sequences used for this study are listed in Supplementary Table S3.

### 2.6 Immunoblotting analysis

Immunoblotting was performed as described previously (Xie et al., 2015). The primary antibodies included total‐IKKβ, phosphorylation‐IKKα/β, total and phosphorylation‐IκBα, total and phosphorylation P65 (Cell Signaling Technology, MA, USA), SCD1, GLUT2, PPARγ, and β‐actin (Santa Cruz, CA, USA). Image J software was used to analyze the intensity of protein expression.

### 2.7 Hematoxylin–eosin staining

Hematoxylin–eosin (HE) staining was performed following the published protocol (Teng et al., 2018). All the images were obtained using a microscope (LEICA DM500, USA). Image J software is used to count liver adipose‐infiltrating cells.

### 2.8 Statistical analysis

The statistic results are presented as mean ± SEM. Graph Pad Prism 5 software is used for statistical analysis and mapping. Multiple groups were compared by one‐way or two‐way ANOVA and Tukey's test. The Student's *t*‐test was conducted to determine the significant differences between two specific groups. *p* < .05 is considered as statistically significant difference.

## 3 RESULTS

### 3.1 GT and Ex ameliorated obese complication in HF mice

**FIGURE 1:**
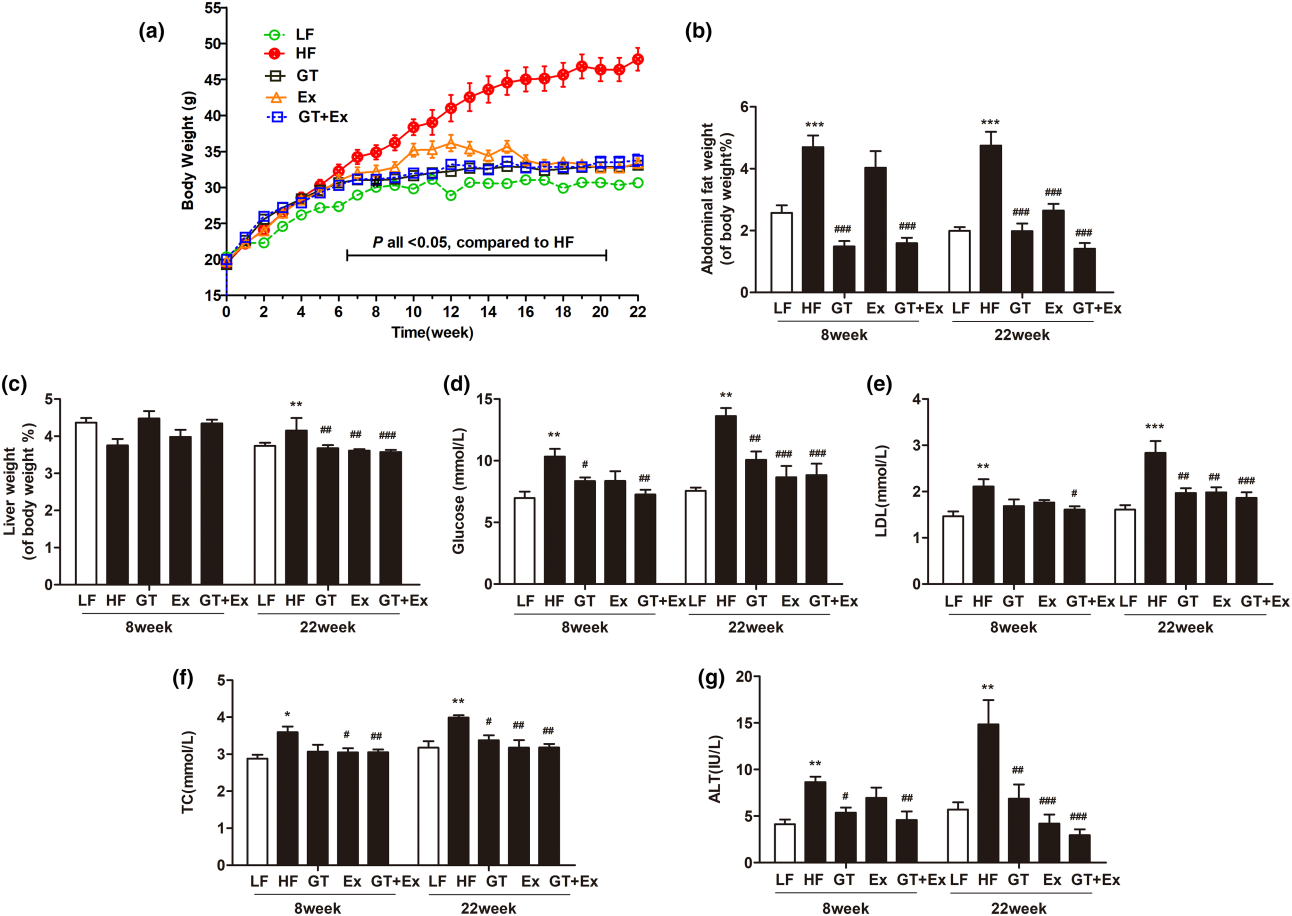
GT and Ex improved symptoms of obesity complications in HF C57BL/6J mice. Body weight (a), abdominal fat weight‐to‐body weight ratio (b), liver weight‐to‐body weight ratio (c), glucose (d), LDL (e), TC (f), and ALT (g) were measured at 8‐ and 22‐week intervention, respectively. *p < .05, **p < .01, and ***p < .001 compared to LF; and ^#^ *p* < .05, ^##^ *p* < .01, and ^###^ *p* < .001 compared to HF (*n* = 6, mean ± SEM). ALT, alanine aminotransferase activity; GT, Yunkang 10 Green Tea; LDL, low‐density lipoprotein; TC, total cholesterol.

**FIGURE 2:**
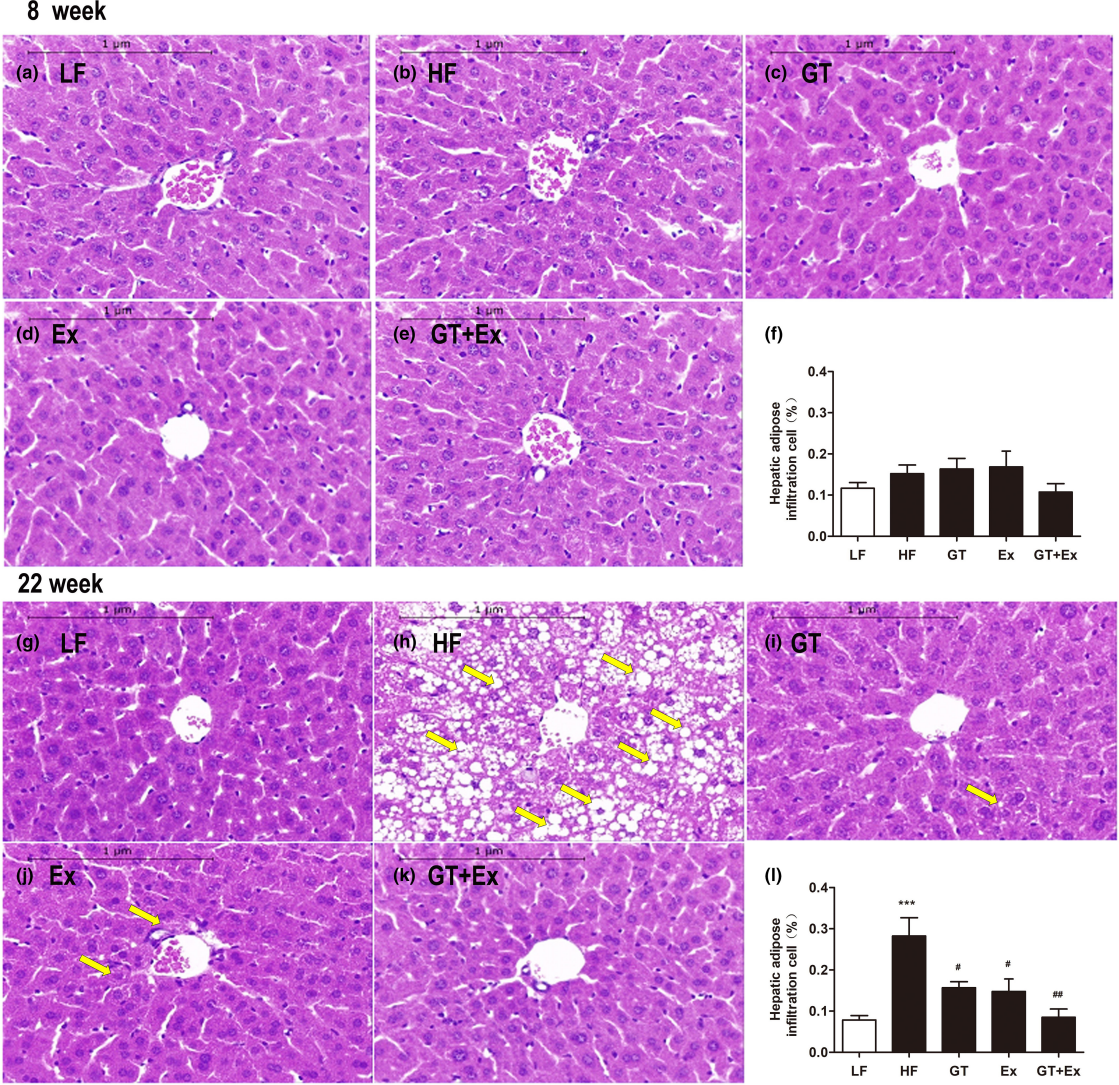
GT and Ex ameliorated fatty liver in HF C57BL/6J mice. The HE staining of liver sections of LF groups (a, g), HF groups (b, h), HF supplement GT groups (c, i), HF with Ex groups (d, j), HF with GT + Ex groups (e, k), and statistic results of hepatic adipose infiltration cell (f, l) were presented at 8‐ and 22‐week intervention, respectively. ***p < .001 compared to LF; and ^#^ *p* < .05 and ^##^ *p* < .01 compared to HF. Yellow arrow pointing toward fat deposition, five images per section and three sections per mice were randomly selected for statistical analysis, *n* = 3, mean ± SEM.

There was no significant change in the food intake of mice during the experiment (Figure 1a). Eight weeks or 22 weeks of high‐fat diet feeding significantly increased C57BL/6J mice body weight, percentage of abdominal fat weight‐to‐body weight ratio, serum glucose, TC, LDL, and activity of ALT (Figure 1b–h). Ex alone for 8 weeks just averted body weight and TC increase (Figure 1b,g). GT alone for 8 weeks significantly prevented the increases in body weight, percentage of abdominal fat weight‐to‐body weight ratio, glucose, and ALT activity. However, intervention with GT + Ex for 8 weeks exhibited synergistic effects and prevented all these index increases (Figure 1b–h). HF feeding of GT, Ex, and GT + Ex treatment for 8 weeks did not alter liver weight‐to‐body weight ratio (Figure 1c), while 22‐week intervention with GT, Ex, and GT + Ex improved all indexes (Figure 1b–h). Our data indicated that long‐term intervention exhibited more profound preventive effects than short term. Liver tissue sections showed the same physiological structures (Figure 2a–e) and existed little amount of adipose infiltration cells (Figure 2f) from all 8‐week intervention groups of mice. However, 22‐week HF‐feeding mice displayed aberrantly fatty hepatocytes with high volumes of lipid droplets. Intervention alone or in combination can maintain normal liver structure and reduce the deposition of fat droplets in hepatocytes (Figure 2g–l).

### 3.2 GT and Ex ameliorated liver inflammation in HF mice

**FIGURE 3:**
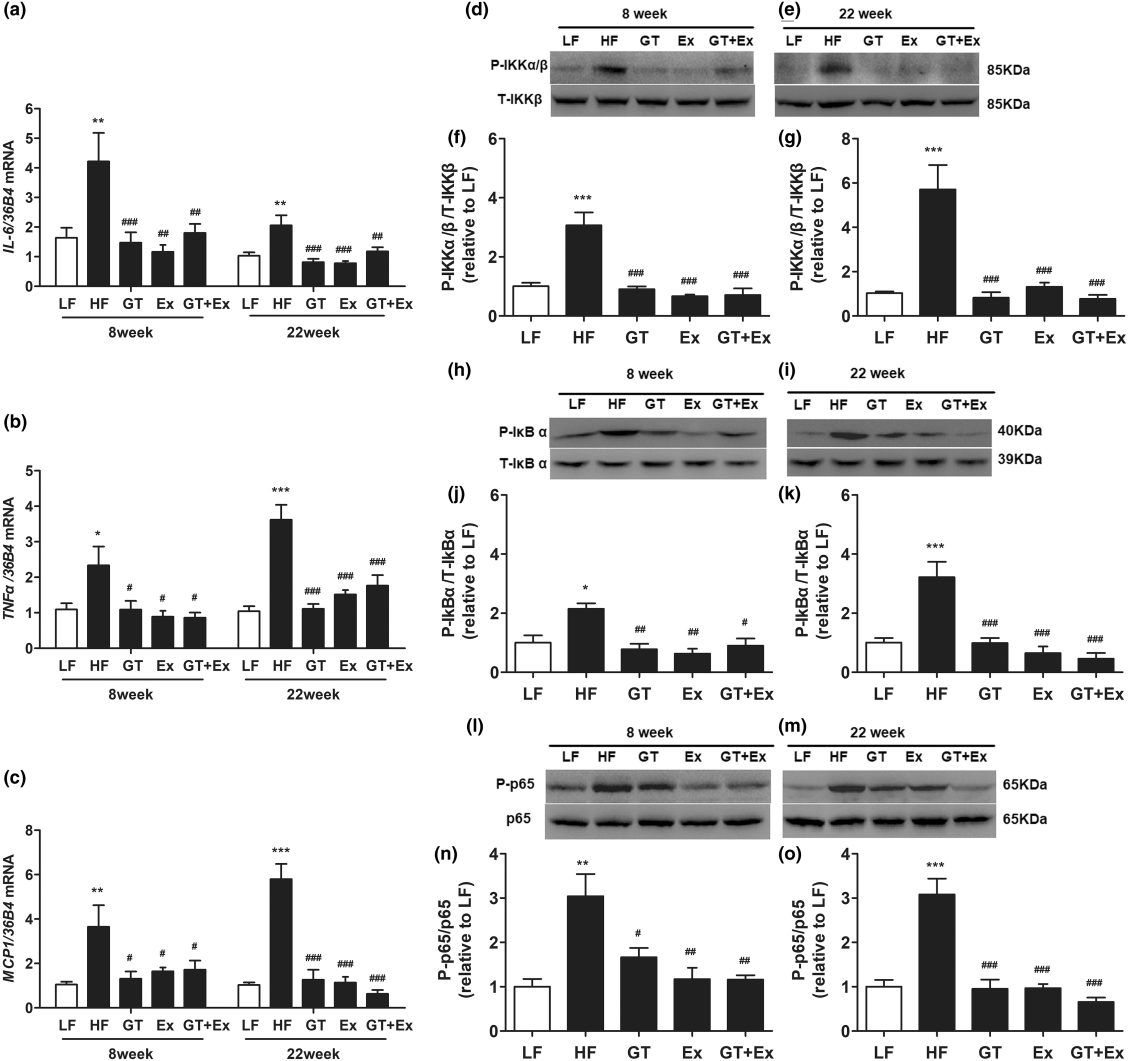
GT and Ex inhibited inflammation and NFκB activation in the liver of HF C57BL/6J mice. The mRNA expression of *IL‐6* (a), *TNFα* (b), and *MCP1* (c) in the liver tissues was quantified by real‐time PCR at 8 and 22 weeks of intervention, respectively. The representing images of protein expression of total IKKβ and phosphorylated IKKα/β (d, e), and statistical results of d (f) and e (g); total and phosphorylated IκBα (h, i), and statistical results of h (j) and i (k); total and phosphorylated P65 (l, m), and statistical results of l (n) and m (o) in the liver tissues at 8‐ and 22‐week intervention, respectively. *p < .05, **p < .01, and ***p < .001 compared to LF; and ^#^ *p* < .05, ^##^ *p* < .01, and ^###^ *p* < .001 compared to HF (*n* = 3–6, mean ± SEM). IL‐6, interleukin 6; MCP‐1, monocyte chemotactic protein‐1; NF‐κB, nuclear factor kappa light chain enhancer of activated B cells; TNFα, tumor necrosis factor α.

Compared with LF mice, the proinflammatory cytokines *IL‐6*, *TNF‐α*, and *MCP‐1* in the liver of HF mice were significantly upregulated. However, both GT and Ex alone and combination of both significantly inhibited the increase of cytokine gene expression in liver tissue (Figure 3a–c). The phosphorylation of IKK and IκBα is the core process of activating NF‐κB pathway. The immunoblotting showed that the phosphorylation of IKKα/β, IκBα, and P65 protein was all dramatically increased in the HF mice liver compared with LF mice. Intervention with GT, Ex, and GT + Ex for both 8 weeks and 22 weeks dramatically prevented these phosphorylated protein increases compared with HF mice (Figure 3d–o).

**FIGURE 4:**
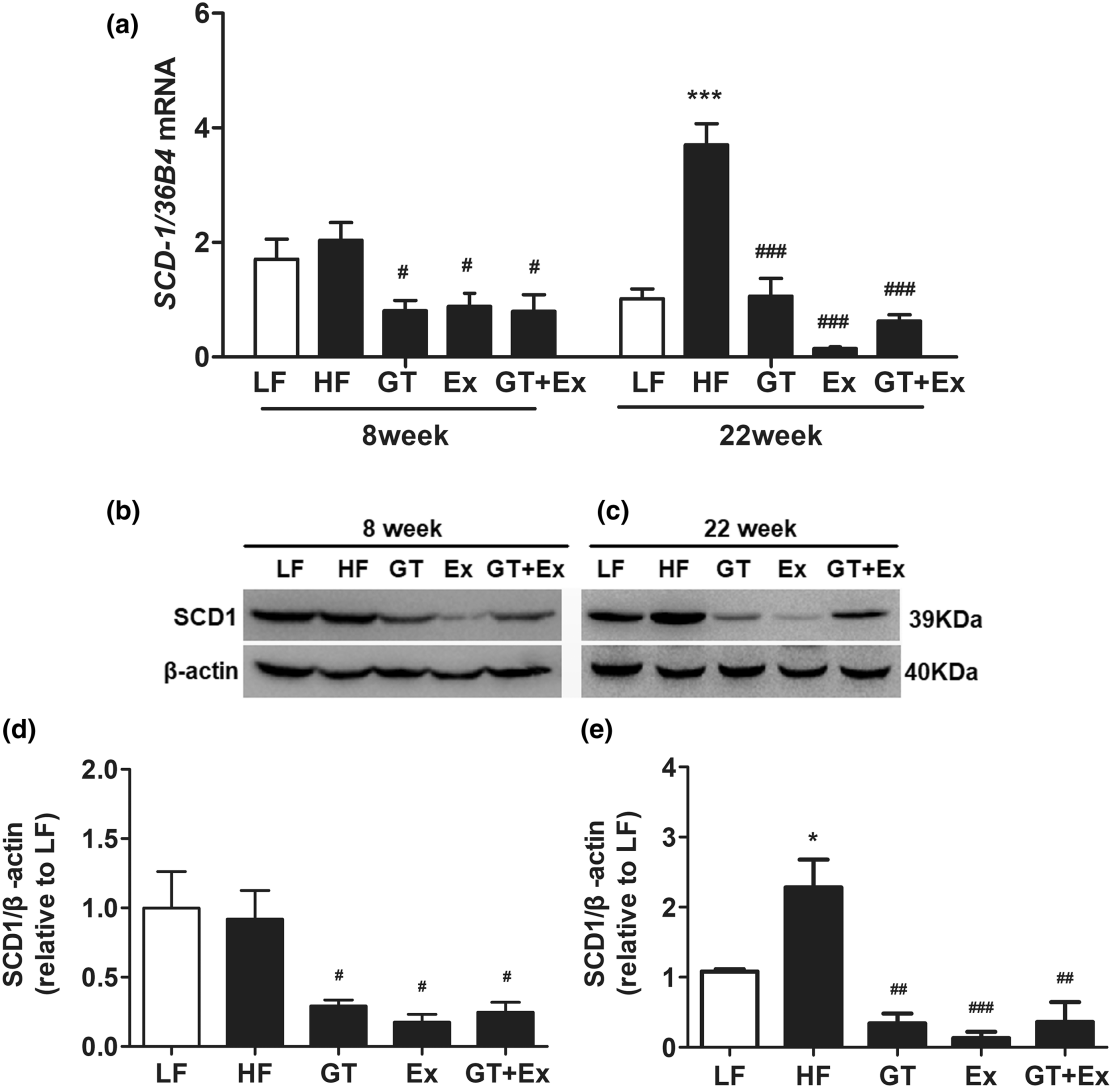
GT and Ex inhibited SCD1 gene and protein expression in the liver of HF C57BL/6J mice. The mRNA expression of *SCD1* (a), representing images of protein expression of SCD1 (b, c), and statistical results of B (d) and C (e) were presented in the liver tissues at 8‐ and 22‐week intervention, respectively. *p < .05 and ***p < .001 compared to LF; and ^#^ *p* < .05, ^##^ *p* < .01, and ^###^ *p* < .001 compared to HF (*n* = 3, mean ± SEM). SCD1, stearoyl CoA desaturase 1.

The current data showed that the phenotype of fatty liver was not altered when the mice were fed HF for 8 weeks (Figure 2a–f). However, proinflammatory cytokines IL‐6, TNF‐α, and MCP1 were significantly upregulated by 8‐week HF feeding (Figure 3a–c). After being fed HF for 8 weeks, the mRNA and protein expression of SCD1 did not change in the livers of HF mice compared with LF mice. However, in an 8‐week intervention with GT, Ex, and GT + Ex, the mRNA and protein expression of SCD1 were significantly downregulated (Figure 4a,b,d). HF feeding for 22 weeks significantly upregulated the SCD1 expression in the liver of HF group mice compared with LF group mice (Figure 4a,c,e). However, the increase of SCD1 gene and protein expression was significantly prevented by GT, Ex, and GT + Ex intervention (Figure 4a,c,e). Our data further found that either GT or Ex prevented both mRNA and protein expression of SCD1 in the liver of HF mice (Figure 4a–e).

We tested the other critical lipid synthesis‐related gene expression, FAS, ACC‐1, and SREBP1, in the liver at 8‐week intervention. As shown in Figure S1, compared with LF group mice, the mRNA expression of *FAS, ACC‐1*, and *SREBP1* in the liver of HF group mice was significantly upregulated. Intervention with GT or Ex or GT + Ex significantly prevented the increases of those gene expression in liver tissue. Additionally, GT + Ex showed synergistic effect to prevent the increase of these genes.

### 3.3 GT and Ex accelerated glucose metabolism in HF mice

**FIGURE 5:**
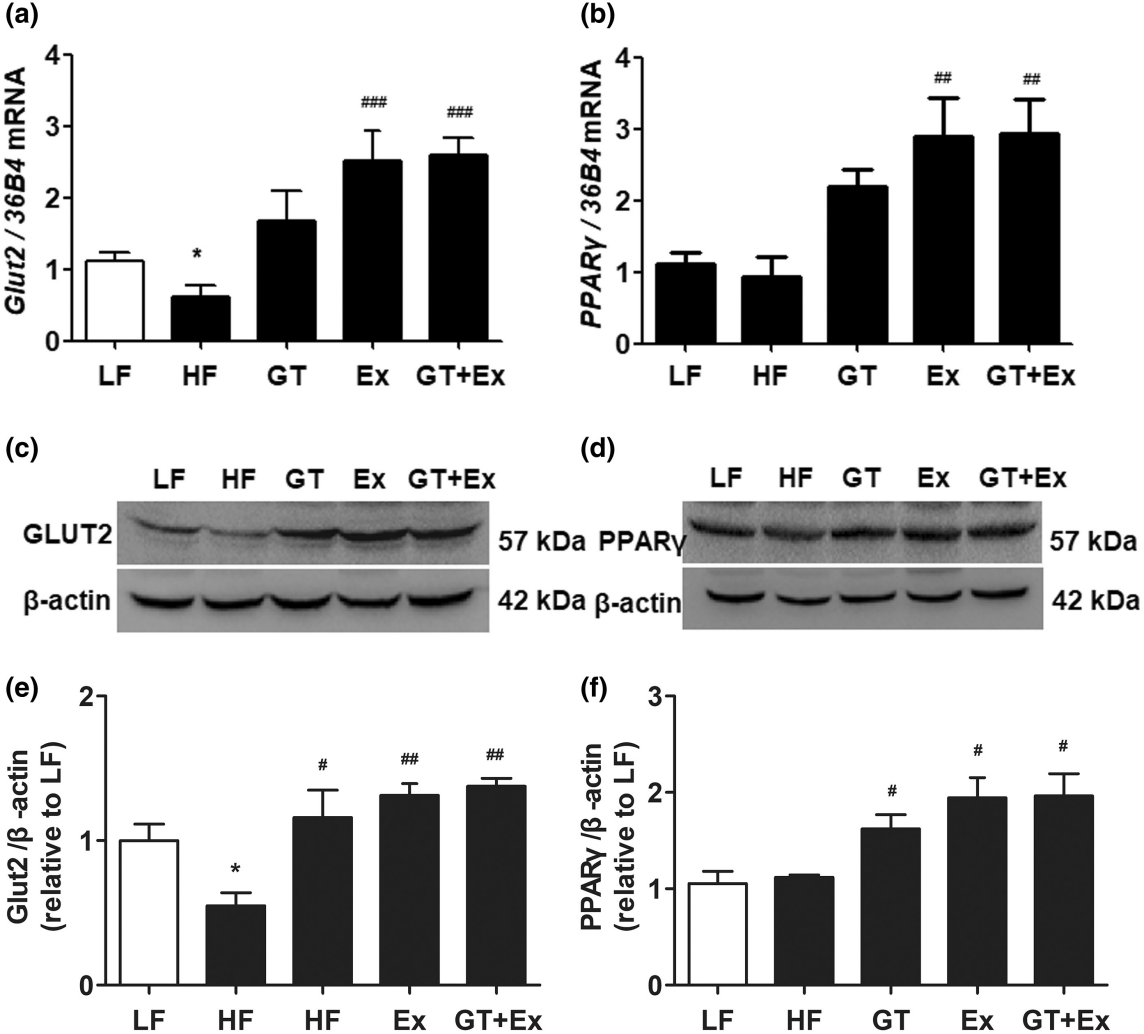
GT and Ex increased GLU2 and PPARγ gene and protein expression in the liver of HFD C57BL/6 mice. The mRNA expression of *GLU2* mRNA (a) and *PPARγ* (b), representing images of protein expression of GLU2 (c) and PPARγ (d), statistical results of c (e) and d (f) were presented in the liver tissues at 22‐week intervention, respectively. *p < .05 compared to LF; and ^#^ *p* < .05 and ^##^ *p* < .01 compared to HF (*n* = 3, mean ± SEM). GLU2, glucose transporter 2; PPARγ, peroxisome proliferator‐activated receptor γ.

HF feeding for 22 weeks significantly decreased GLUT2 gene and protein expression in the liver of HF mice. However, a supplement of GT, or Ex or GT + Ex, significantly increased GLUT2 gene and protein expression in the liver (Figure 5a,c,e). Additionally, the gene and protein expression of PPARγ did not alter in the livers of HF group mice compared to that of LF group mice, while GT, Ex, and GT + Ex obliviously increased PPARγ gene and protein expression in the liver of treated group mice (Figure 5b,d,f). Our data further revealed that intervention with GT, Ex, or GT + Ex for 22 weeks significantly upregulated GLUT2 gene and protein expression in the liver of HF mice (Figure 5a,c,e), which may contribute to the amelioration of HF‐induced hepatic steatosis. The present results found that gene and protein expression of PPARγ were increased in the liver of HF mice treated with GT, Ex, and GT + Ex for 22 weeks (Figure 5b,d,f).

## 4 DISCUSSION

**FIGURE 6:**
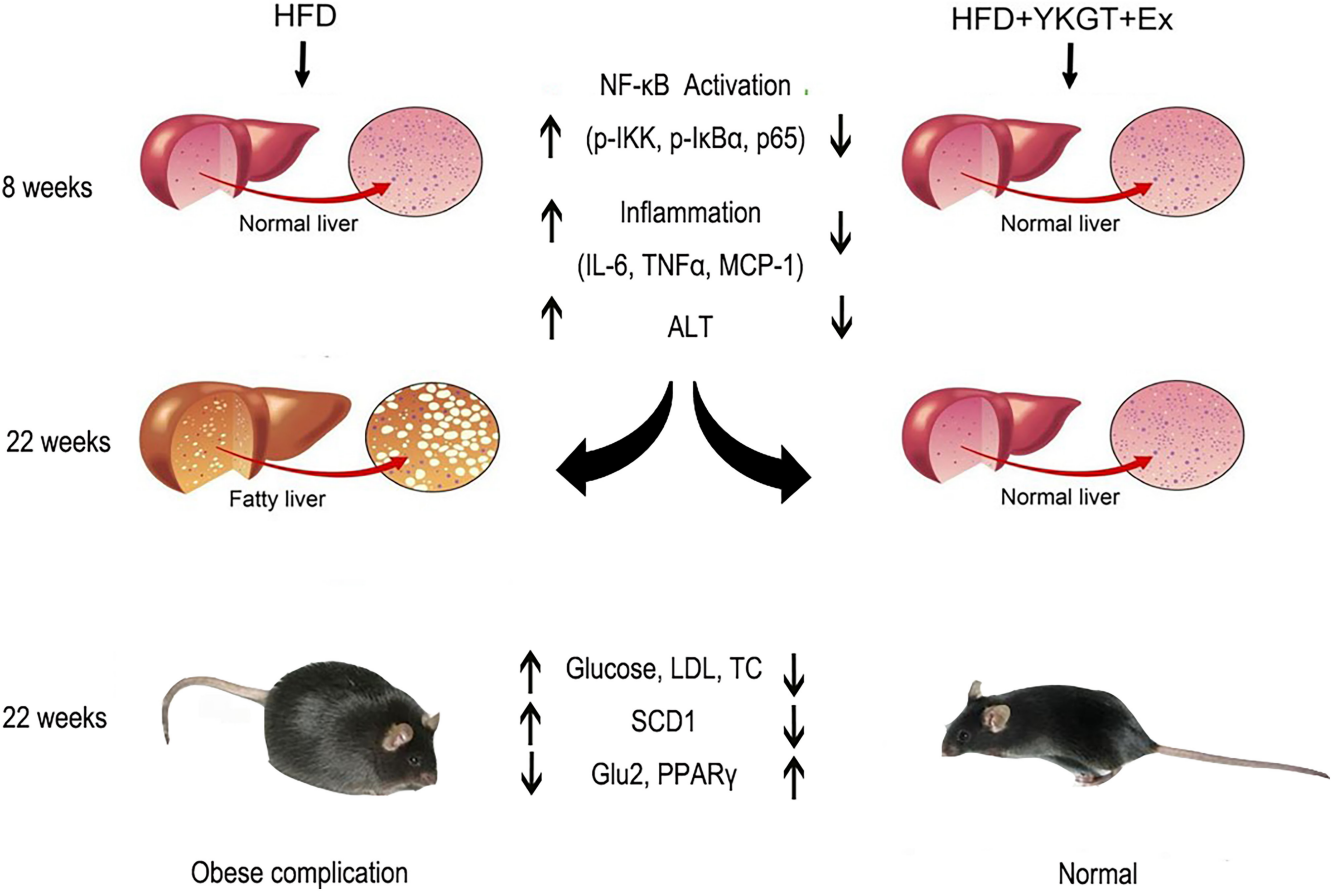
The schematic diagram for this study.

Obesity complications include obesity, dyslipidemia, diabetes, and fatty liver. Inflammation initiates a vicious cycle between obesity and nonalcoholic fatty liver disease (Luo & Lin, 2021). Fatty liver disease is characterized by chronic hepatic inflammation. Recently, Bagheri et al. found that the combination of GT and Ex promotes a further decrease in weight, body mass index, and body fat percentage than exercise alone in inactive overweight women (Bagheri et al., 2020). Khoo et al. also reported that the combination of decaffeinated green tea extract and voluntary exercise synergistically mitigated nonalcoholic fatty liver disease in HF mice (Khoo et al., 2020). The current data indicated that for the 8‐week treatment, exercise did not show obvious effects on decreasing of adipose fat, glucose level, and ALT activity. The GT + EX effects seen appear to be primarily due to GT treatment. However, a long‐term 22‐week intervention exhibited profound preventive effect than that of the 8‐week treatment. The diagram of this study is shown in Figure 6.

Indeed, Yamaguchi et al. found that HF elevated plasma IL‐6, and blockade of IL‐6 signaling ameliorated systemic insulin resistance and improved hepatic steatosis in HF‐fed mice (Yamaguchi et al., 2015). NF‐κB contains multiple protein complexes that control cytokine production. The phosphorylation of IKK and IκBα is a key factor for activation of NF‐κB pathway (Chen et al., 2021). Our results showed that GT, Ex, and GT + Ex all decreased the phosphorylation of IKKα/β and IκBα, and thus inhibited the transcript of proinflammatory cytokines at both short‐ and long‐term intervention. Moreover, the phosphorylated NFκB p65 subunit was significantly downregulated by GT, Ex, and GT + Ex at 8‐ and 22‐week intervention. Li et al. reported that GTE decreased the phosphorylation of the NFκB p65 subunit and alleviated nonalcoholic steatohepatitis NASH in HF mice (Li et al., 2017). Our data further found that GT, Ex, and GT + Ex ameliorated hepatic steatosis by inhibiting NFκB activation in the liver of HF mice.

The accumulation of excess fat in the liver is a primary cause of hepatic steatosis. SCD1 is a key regulator for de novo lipogenesis in the liver. SCD1 is also a central regulator of fuel metabolism and catalyzes the synthesis of monounsaturated fatty acids (MUFA). Variations in SCD1 activity have been associated with obesity, diabetes, or inflammation, and SCD1 increased levels may represent lipid storage activity in omental adipose tissue (Olga et al., 2021). Zhou et al. found that the aqueous extract of postfermented tea reversed the hepatic steatosis of hyperlipidemia rats by downregulating the hepatic SCD1 gene expression in HF‐fed rats (Zhou et al., 2014). Yang et al. reported that long‐term DHA supplementation (DHA), treadmill running (EX), and DHA + EX up to 18 months all decreased Scd1 expression and ameliorated fatty liver in HF C57BL/6J mice. Consistent with previous reports, our study showed that an increase in inflammatory cytokines is a preceding inducer in the development of hepatic steatosis, and long‐term GT and Ex or combination inhibit the expression of SCD‐1 in the liver of HF mice. But, as shown in Figures 2 and 4, there were no changes in SCD1 expression between the HF and LF groups. We speculate that 8 weeks did not significantly induce fat accumulation in the liver of mice. Moreover, GLUT2 is the predominant glucose transporter in hepatocytes, which plays a key role in protecting the liver from inflammation and fatty liver (Wu et al., 2020). Few studies have investigated the exact signaling molecules that may explain the mechanism following exercise training with or without longer term GTE intake in human and animal studies (Hodgson et al., 2013). GLUT2 represents the main gate of glucose uptake by the liver. Ahmed et al. reported that the mutation of Soat2 reduces liver steatosis by regulating the GLUT2 in female mice (Ahmed et al., 2019). Green tea polyphenol extract upregulates the GLUT2 expression in the liver of rats fed a high fructose diet (Cao et al., 2007). Importantly, we further expanded our mechanism exploration in this study and found that long‐term tea drinking and exercise may have synergistic health benefits. PPARγ has crucial roles in adipogenesis and lipid accumulation within adipocytes (Park et al., 2016). Previous research (Li et al., 2018) reports that EGCG inhibited the expression of genes involved in the synthesis of de novo fatty acids, which include *acc1*, *fas*, *scd1*, and *srebp1*. However, both exercise and tea drinking, as well as their combination, increased the expression of PPARγ at 22 weeks. The expression changes of PPARγ are different from previous studies. The possible reason may be due to the different physiological reactions between direct consumption of green tea powder and feeding EGCG. Multiple studies have shown that PPARγ regulates downstream genes, reduces the accumulation of triglycerides in cells, increases cholesterol efflux from liver tissue, and prevents steatosis (Chyau et al., 2020; Li et al., 2023). In addition, PPAR γ also regulated inflammatory factors in various ways to suppress inflammation in NAFLD, which included terminating NF‐ κB P65 transcription and downregulating IL‐6, TNF‐α, IL‐4, IL‐10, and IL‐2 inflammatory factors to improve inflammation (Abduh et al., 2023; Gross et al., 2017). Our data suggested that the GT + EX combined intervention for 22 weeks inhibited the activation of NF‐κB pathway, decreased the expression of proinflammatory cytokines, and consequently improved hepatic statuses.

Collectively, our study suggested that GT supplementation with exercise effectively relieves hepatic steatosis and obesity complications in HF mice by ameliorating hepatic inflammation, reducing lipid synthesis, and accelerating glucose transport and metabolism. Our results found that long‐term green tea drinking and regular aerobic exercise are good habits for preventing hepatic steatosis and obesity complications in the human population.

## AUTHOR CONTRIBUTIONS

**Ruru Wang:** Writing – original draft (lead). **Mingxing Gu:** Investigation (equal). **Yanzhong Zhang:** Software (equal). **Qinglin Zhong:** Data curation (equal). **Linbo Chen:** Methodology (equal). **Daxiang Li:** Resources (equal). **Zhongwen Xie:** Supervision (equal).

## FUNDING INFORMATION

This work was supported by the key joint grant for regional innovation from the National Natural Science Foundation of China to Z.X [Grant Number U19A2034]; a key grant for the University Synergy Innovation Program of Anhui Province to Z.X [Grant Number GXXT‐2019‐49]; an open grant from State Key Laboratory of Tea Plant Biology and Utilization to Y.Z [Grant Number SKLTOF20170102]; and a grant from China Agriculture Research System of MOF and MARA [CARS‐19].

## CONFLICT OF INTEREST STATEMENT

The authors declare that they have no known competing financial interests or personal relationships that could have appeared to influence the work reported in this paper.

## ETHICS STATEMENT

The animal experiment procedures implemented in this study follow the guidelines of the Animal Care and Use Committee (IACUC) of Anhui Agricultural University, with the ethical approval code of AHAU 2016–002.

## Supporting information


Data S1.

## Data Availability

The original data and supplementary materials provided in this article can be obtained from the corresponding authors.
