# Supplementary material for: Long‐term drinking of green tea combined with exercise improves hepatic steatosis and obesity in male mice induced by high‐fat diet
Source: Food Sci Nutr. 2023 Dec 21;12(2):776–85. doi: 10.1002/fsn3.3773 (PMC10867457; doi:10.1002/fsn3.3773)
Supplement: Supplementary file 1 — Data S1. [file FSN3-12-776-s001.doc]

Supplementary Material

# Supplementary Tables

## Table S1: The chemical composition of HF (TP23300) and LF (TP23303)

| Composition | TP23300 (60% HF) g (%) | TP23303 (10% LF) g (%) |
| --- | --- | --- |
| Protein (casein, L-cystine) | 276 | 194 |
| Carbohydrate (dextrin, sucrose) | 250 | / |
| Carbohydrate (dextrin, sucrose, corn starch) | / | 673 |
| Fat (soybean oil, lard) | 341 | 40 |
| Fiber (Cellulose) | 68 | 48 |
| Mineral and vitamin mixture | 65 | 45 |
| Antioxidant (TBHQ) | 0.07 | 0.01 |
| Total | 1000 | 1000 |
|  | TP23300 (60% HFD) | TP23303 (10% LFD) |
| Energy, kcal/g | 5.1 | 3.6 |
| % Kcal from protein | 19% | 19% |
| % Kcal from carbohydrate | 21% | 71% |
| % Kcal from fat | 60% | 10% |
| Total | 100% | 100% |

# Table S2: The movement speed and time of mice during the experiment

| Weeks | Speed (m/min) | Time (min) |
| --- | --- | --- |
| 1 | 6 | 30 |
| 2-3 | 8 | 40 |
| 4-5 | 8 | 70 |
| 6-22 | 8 | 110 |

| Number | Gene | Forward primer (5'-3') | Reverse primer (5'-3') |
| --- | --- | --- | --- |
| 1 | *36B4* | CCCTGAAGTGCTCGACATCA | TGCGGACACCCTCCAGAA |
| 2 | *IL-6* | CTGCAAGAGACTTCCATCCAGTT | GGCTGCCCCGACTACGT |
| 3 | *TNFα* | GGCTGCCCCGACTACGT | ACTTTCTCCTGGTATGAGATAGCAAA |
| 4 | *MCP1* | CTTCCTCCACCACCATGCA | CCAGCCGGCAACTGTGA |
| 5 | *GLUT2* | TCAGAAGACAAGATCACCGGA | GCTGGTGTGACTGTAAGTGGG |
| 6 | *SCD1* | TCCTCCTTGGATTGTGTAGAAACTT | AATGTCAGAAGAAATCAGGTGGGTA |
| 7 | *PPARγ* | GAAAGACAACGGACAAATCACCAT | CGGCTTCTACGGATCGAAACTG |
| 8 | *ACC1* | AGGAGGGAAAGGGATCAGAAA AG | CAGAGCAGTCACGACCAAACAAA |
| 9 | *SREBF* | AGTCCAGCCTTTGAGGATAGCC | CCGTAGCATCAGAGGGAGTGAG |
| 10 | *FAS* | CGTGTGACCGCCATCTATATCG | TGAGGTTGCTGTCGTCTGTAGTCTT |

# Table S3: Primer sequences used for RT-PCR gene expression experiment.


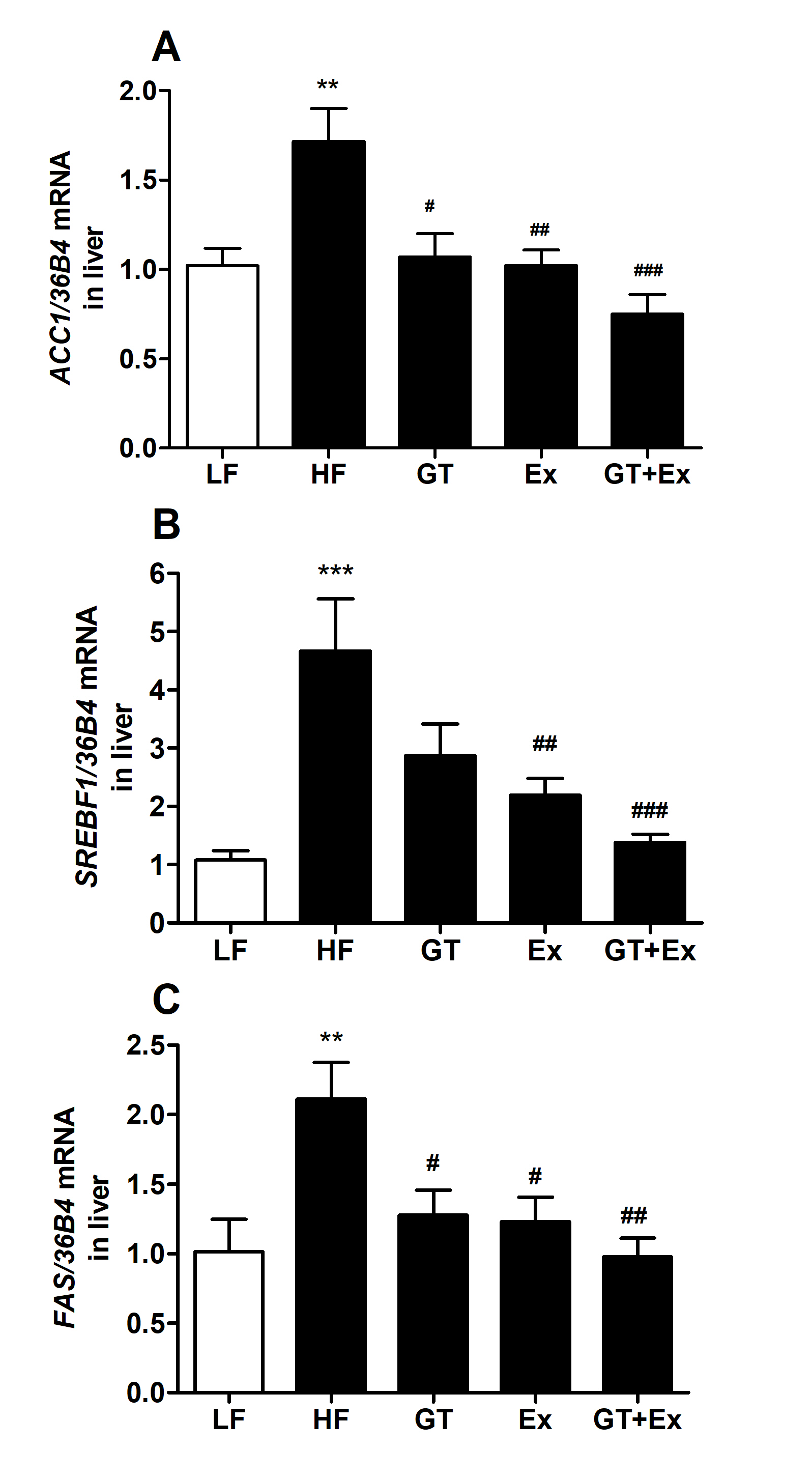


**Figure S1:** The mRNA expression of *ACC 1* (A), *SREBF1* (B), *FAS*(C) in the liver tissues were quantified by Real Time PCR at 8-week intervention, respectively. *P<0.05, **P<0.01, compared to LF; and ^#^P<0.05, ^##^P<0.01, ^###^P<0.001 compared to HF (n=4-6, mean ± SEM). ACC1, Acetyl-CoA carboxylase; SREBF1, sterol regulatory element-1; FAS, fas cell surface death receptor.
